# Dutch Translation and Psychometric Testing of the 9-Item Shared Decision Making Questionnaire (SDM-Q-9) and Shared Decision Making Questionnaire-Physician Version (SDM-Q-Doc) in Primary and Secondary Care

**DOI:** 10.1371/journal.pone.0132158

**Published:** 2015-07-07

**Authors:** Sumayah Rodenburg-Vandenbussche, Arwen H. Pieterse, Pieter M. Kroonenberg, Isabelle Scholl, Trudy van der Weijden, Gre P. M. Luyten, Roy F. P. M. Kruitwagen, Henk den Ouden, Ingrid V. E. Carlier, Irene M. van Vliet, Frans G. Zitman, Anne M. Stiggelbout

**Affiliations:** 1 Department of Psychiatry, Leiden University Medical Centre, Leiden, The Netherlands; 2 Department of Medical Decision Making, Leiden University Medical Centre, Leiden, The Netherlands; 3 Department of Child and Family Studies, Leiden University, Leiden, The Netherlands; 4 Department of Medical Psychology, University Medical Centre Hamburg-Eppendorf, Hamburg, Germany; 5 Department of Family Medicine, Maastricht University, School CAPHRI, Maastricht, The Netherlands; 6 Department of Ophthalmology, Leiden University Medical Centre, Leiden, The Netherlands; 7 Department of Gynaecology, Maastricht University, Maastricht, The Netherlands; 8 Julius Centre for Health Sciences and Primary Care, University Medical Centre Utrecht, Utrecht, The Netherlands; institute of Health Policy and Management, NETHERLANDS

## Abstract

**Purpose:**

The SDM-Q-9 and SDM-Q-Doc measure patient and physician perception of the extent of shared decision making (SDM) during a physician-patient consultation. So far, no self-report instrument for SDM was available in Dutch, and validation of the scales in other languages has been limited. The aim of this study was to translate both scales into Dutch and assess their psychometric characteristics.

**Methods:**

Participants were patients and their treating physicians (general practitioners and medical specialists). Patients (*N* = 182) rated their consultation using the SDM-Q-9, 43 physicians rated their consultations using the SDM-Q-Doc (*N* = 201). Acceptability, reliability (internal consistency), and the factorial structure of the instruments were determined. For convergent validity the CPS_post_ was used.

**Results:**

Reliabilities of both scales were high (alpha SDM-Q-9 0.88; SDM-Q-Doc 0.87). The SDM-Q-9 and SDM-Q-Doc total scores correlated as expected with the CPS_post_ (SDM-Q-9: *r* = 0.29; SDM-Q-Doc: *r* = 0.48) and were significantly different between the CPS_post_ categories, with lowest mean scores when the physician made the decision alone. Principal Component Analyses showed a two-component model for each scale. A confirmatory factor analysis yielded a mediocre, but acceptable, one-factor model, if Item 1 was excluded; for both scales the best indices of fit were obtained for a one-factor solution, if both Items 1 and 9 were excluded.

**Conclusion:**

The Dutch SDM-Q-9 and SDM-Q-Doc demonstrate good acceptance and reliability; they correlated as expected with the CPS_post_ and are suitable for use in Dutch primary and specialised care. Although the best model fit was found when excluding Items 1 and 9, we believe these items address important aspects of SDM. Therefore, also based on the coherence with theory and comparability with other studies, we suggest keeping all nine items of the scale. Further research on the SDM-concept in patients and physicians, in different clinical settings and different countries, is necessary to gain a better understanding of the SDM-construct and its measurement.

## Introduction

In recent years, there has been an increasing emphasis on the role of patients’ preferences and of shared decision making (SDM) in treatment decision making. There is a growing recognition that this sharing is important. In partnership with their clinicians, patients are encouraged to consider the likely harms and benefits of available treatment options, communicate their preferences, and select the option that best fits these [1]. SDM helps to ensure that treatment decisions reflect patient preferences so that patient experiences of care as well as treatment outcomes may improve [2]. Thus SDM is a critical part of quality care and should be one of the principles for good clinical practice [3–5]. Although great efforts are made to promote shared decision making, the measurement of its construct is challenging [6] and evidence on its impact remains sparse [7]. Reliable and valid instruments are needed for studies that assess the effectiveness of SDM. In addition, these can help gain a better understanding of the concept of SDM and its correlates. Furthermore such instruments will help to facilitate the development, implementation, and evaluation of decision making interventions in clinical practice.

An important self-report instrument developed to measure the process of shared decision making as perceived by the patient is the nine-item Shared Decision Making Questionnaire (SDM-Q-9) [6]. The original German SDM-Q was developed building on Elwyn’s model of competences for involving patients, and on additional psychological theories [6,8]. This 24-item questionnaire, underwent a major revision and was reduced to a 9-item scale, the SDM-Q-9. The scale had a high internal consistency, high item discriminations, and showed high face and factorial validity [6]. In addition to this patient version a German physician-version, the SDM-Q-Doc, was constructed as well, by rephrasing the questions of the original SDM-Q-9. The SDM-Q-Doc showed good internal consistency and acceptable to good item discriminations, indicating a good reliability of the scale [9].

Over the last years the SDM-Q-9 has become a frequently used instrument to measure SDM in clinical practice, and has been translated into several languages, including English [6, 10], Spanish [11], and French, Italian, Chinese, Japanese, Korean, Persian and Hebrew (I Scholl, personal communication, 2014). Untill now, no Dutch version was available.

However, validation of the various translations was limited and little has been done to establish the convergent validity of the scale [10,12]. To our knowledge, only the Spanish SDM-Q-9 was officially translated,validated (mostly with respect to internal consistency) and published [11]. It showed some differences in the factor structure with the original German scale. Furthermore, most of the validation studies of the SDM-Q-9 have been carried out using patients in primary care. The internal consistency of the German SDM-Q-Doc was tested in a primary and a specialised care sample, but there has been no further validation of this scale yet.

Therefore, the present study set out to translate the SDM-Q-9 and the SDM-Q-Doc into Dutch and to evaluate their psychometric properties using Dutch primary and specialised care samples of patients and their treating physicians from different medical specialities.

## Method

### Ethics statement

The study was approved by the Medical Ethics Committee of the University Medical Centre Utrecht, the Medical Ethics Committee of Maastricht University and Medical Ethics Committee of the Leiden University Medical Centre (Reference number P12.043). All participants provided their written consent before filling in the questionnaires.

### Sample

The study sample was composed of outpatients and their treating medical specialists at the Departments of Psychiatry and of Ophthalmology of Leiden University Medical Centre; the Departments of Gynaecology and of Oncology (breast cancer) of the Maastricht University Medical Centre, and Type 2 Diabetes patients and their general practitioners, participating in the OPTIMAL study of the Julius Centre for Health Sciences and Primary Care, Utrecht University Medical Centre.

We aimed at a sample of 180 patients, based on the heuristic of 15–20 patients per item of the questionnaire. We intended to recruit five physicians from every department and each participating physician was asked to collect data from 10 patients who met the following eligibility criteria: a) above 17 years of age; b) able to speak and read Dutch; and c) facing a decision regarding the health problem for which they visited their physician.

### Procedures

To establish the study sample, the heads of each participating department agreed to inform and recruit physicians of their own departments (GPs, specialists, and residents). Physicians were informed about the project and asked to recruit 10 of their consulting outpatients.

Physicians were instructed to inform their eligible patients about the study and to ask informed consent for their participation. For each participating patient, physicians were asked to complete after the consultation: the SDM-Q-Doc, including two open-ended questions on what health problem was the subject of the consultation and which decision was made [6], and Kasper et al.’s single-item modification of the Control Preferences Scale (CPS), CPS_post_ [13] (see Measures, paragraph 2.3.3). Physicians also completed a (once only) short questionnaire on their demographic characteristics.

Patients willing to participate first signed an informed consent form. Immediately after the consultation, patients were asked to rate the extent to which they felt involved in decision making by filling out the SDM-Q-9 (including the two open-ended questions), the CPS_post_, and questions on demographic characteristics.

Both patients and physicians were asked to fill in their own name and the name of their patient/physician. This information was only used to link patient and physician data.

### Measures

#### SDM-Q-9 and SDM-Q-Doc

The SDM-Q-9 and the SDM-Q-Doc measure the views of respectively the patient and physician on the decision-making process in a consultation. The nine items of the scale each describe a different step of the SDM process, for example “My physician made clear that a decision needs to be made” and “My physician and I selected a treatment option together” [8]. All items are scored on six-point Likert scales ranging from 0 (“completely disagree”) to 5 (“completely agree”). The aggregated scores over all items of the SMD-Q-9 lead to a total raw score between 0 and 45, with 0 indicating the lowest and 45 indicating the highest level of perceived SDM.

#### Translation of the questionnaires

First, two native Dutch speakers with fluent command of the German language independently translated the original German versions of the SDM-Q-9 and SDM-Q-Doc into Dutch. These translations were then discussed in a consensus meeting, consulting the English version [6] in case of ambiguity (see S1 Appendix). The agreed upon Dutch versions were then back-translated into German by two native German speakers with fluent command of the Dutch language. The original questionnaires and the back-translations were compared and discrepancies were resolved between four members of the research team (among whom IS and AS). At the end of the translation process, both versions were presented to several clinicians for their opinion. Because the clinicians found that the phrasing of the words “ausdrücklich mittgeteilt” (“uitdrukkelijk medegedeeld”) was too strong in Dutch, and that they are seldom used in Dutch clinical practice, we preferred to use „made clear”(„duidelijk gemaakt“). In addition “mitgeteilt” sounds too formal in Dutch, therefore we chose “told” (“verteld”), as in the English translation. This was discussed and agreed upon by the research team, resulting in the final version. See S1 Appendix for the description of the items of the SDM-Q-9 and SDM-Q-Doc in English.

#### CPS_post_

As a gold standard for measuring the perceived level of involvement is lacking, we chose a modified version of the Control Preferences Scale, the CPS_post_ for comparison. The CPS_post_ is a five-point Likert scale formulated to measure the experienced role in the final decision, which has a good reliability and validity [13]. The original Control Preferences Scale, was developed by Degner [14] to measure preference for involvement and is one of the most commonly used instruments to assess someone's preferred decisional role. Modifications of the CPS as a single five-point Likert scale to measure someone's experienced role have been used in different studies, showing good reliability and validity [13, 15, 16]. An example is Kasper et al’s (2011) CPS_post_, which was used in a validation study on inter-relating measures for SDM. The authors reported a moderate association between the CPS_post_ and SDM-Q-9, a more autonomous role (CPSpost) was associated with more involvement as reported on the SDM-Q- 9. For this study a physician's version was made by rephrasing the CPS_post_ item.

The CPS_post_ presents subjects with a choice of five alternative decisional roles. Patients/physicians were asked to indicate who made the decision: 1:“I made the decision alone”, 2: “I made the decision alone considering my physician’s opinion /patient’s preferences”, 3:”I shared the decision with my physician /patient”, 4:”My physician /patient decided considering my opinion/preferences” and 5:”My physician /patient made the decision”.

### Statistical analyses

As the summated SDM-Q-9 and SDM-Q-Doc raw scores have an unfamiliar range (0–45), we followed Kriston et al. [6] and rescaled this range to 0–100; the rescaled version is used throughout this paper. First, we investigated the characteristics of the frequency distributions of the total scores (mean, standard deviations, skewness and kurtosis). In addition, acceptance rates of the questionnaire items were assessed as the percentage of participants who were willing to fill out a particular item.

Item difficulties were determined by calculating the mean total score of each item. Low mean scores, below the midpoint (2.5 on a 6-point Likert scale ranging from 0 to 5), can be interpreted as a generally difficult aspect of SDM behaviour to achieve in a consultation.

Secondly, we assessed the internal consistency of the SDM-Q-9 and SDM-Q-Doc with Cronbach’s-alpha [17]. We also determined whether all items were contributing sufficiently to the scales by computing both corrected item-total correlations and the value of Cronbach’s alpha if the item were deleted. Considering that both questionnaires (SDM-Q-9 and SDM-Q-Doc) have proven to be psychometrically sound instruments in several other samples, we expected to find good internal consistency (Cronbach’s alpha>.70) for both scales, comparable to other studies.

We evaluated the SDM-Q-9 for dependencies within consultations by means of intraclass correlation coefficients (ICCs), to determine if we needed to take the hierarchical nature of the data into account (some patients were treated by the same physician). We then evaluated convergent validity of the SDM-Q-9 and the SDM-Q-Doc by using Spearman’s correlation coefficient to assess the association of the total (standardized) SDM scores with the CPS_post_ item treated as an ordinal variable. We also used the CPS_post_ as a variable with five nominal categories and compared the mean SDM scores between the categories using an analysis of variance. With regard to convergent validity, our hypothesis was that the SDM-Q-9 / SDM-Q-Doc would have a significant moderate to good correlation (*r* = 0.40–0.60) with the CPS_post,_ based on Kasper et al.’s (2011) findings. Furthermore we expected the mean scores of the SDM-Q-9 and SDM-Q-Doc to be highest on the CPS_post._, when the decision was considered to be shared.

Next, we carried out a principal component analysis (PCA) to assess the scales’ dimensionalities. We assessed whether additional components could be extracted that would call into question the appropriateness of the single-component hypothesis. To this end, the eigenvalues and the scree plot, as well as the amount of variance accounted for were used.

Given that earlier studies on the SDM-Q-9 and the SDM-Q-Doc showed a one-dimensional structure based on confirmatory factor analyses (CFA)[6, 9], we also performed such analyses using EQS software (Multivariate Software Inc., Encino, California, USA). Since the χ^2^ statistic used to test model fit is highly sensitive to conceptually unrelated technical conditions (like violation of the normality assumption and sample size), the fit of the models was also evaluated by means of descriptive fit indices, such as the comparative fit index (CFI), root mean square error of approximation (RMSEA), and the standardized root mean square residual (SRMR) [18, 19]. The CFA models were regarded as acceptable to good when the fit indices met the following cut off criteria: RMSEA ≤ 0.06; CFI > 0.95 and SRMR ≤ 0.08 [19, 20].

Apart from the confirmatory factor analyses, the statistical analyses were performed using IBM SPSS Statistics Version 20 (IBM, Chicago, IL, USA).

### Post-hoc analyses

As the distribution of the scores on both SDM questionnaires was clearly non-normal, it was decided to carry out a variety of tests, all requiring different assumptions for the dependent variables. Moreover, taking the observed sample distributions as the best approximation to the population distributions, 1000 bootstrap samples were taken from the observed sample distribution, and their associated confidence intervals for the means were calculated.

## Results

### Sample characteristics

A total of 182 patients rated their consultations with 44 physicians. Two-thirds (65%) of the patient sample was female. Their mean age was relatively high, 61 years (*SD* = 15.5), with a range from 19 to 88 (Table 1).

**Table 1:** Sample Characteristics of the Patients.

		N = 182*	in %
**Gender**			
	**Male**	61	34
	**Female**	119	65
**Age** **	**Mean (SD; range)**	60.9(15.5; 19–88)	-
**Health problem**			
	**Type 2 diabetes**	74	41
	**Psychiatric**	36	20
	**Ophthalmic**	36	20
	**Gynaecologic**	24	13
	**Breast Cancer**	12	7

* The sample size varies between 177 and 182 due to missing values.

** As reported on the SDM-Q-9 by participating patients.

Forty-three different physicians (23 specialists and 20 general practitioners) rated 213 consultations with their patients (see Table 2 for numbers by speciality). The physician sample consisted of slightly more men than women (58%). Due to a different procedure for the general practitioner sample, no demographic data other than gender were available for the latter group. The mean age of the specialists was 34 years, ranging from 24 to 60 years. The mean number of patients rated by each physician was five, with values ranging from 1 to 15.

**Table 2:** Sample Characteristics of the Physicians.

		N = 43*	in %
**Gender**			
	**Male**	23	58
	**Female**	17	43
**Age**	**Mean (SD; range)**	34(10.8; 24–60)	-
**Profession**			
	**General practitioner**	20	36
	**Psychiatrist**	11	28
	**Gynaecologist**	7	18
	**Ophthalmologist**	4	18
	**Surgeon (oncologic)**	1	1

* The sample size varies between 40 and 43 due to missing values.

Missing questionnaires of both patients and physicians caused differences in the numbers of participants in the analyses. In some cases, patients completed their questionnaires but their physician did not or vice versa. The reliability analysis and principal component analyses were based on datasets with complete data for all of the nine items (***N***_*pat*_ = 160 and ***N***_doc_ = 201). It was not feasible to obtain rates on how many physicians were asked to participate but declined, because physicians were informed and asked to participate by the head of their department. Additionally, because it was already difficult to get physicians to participate, we did not ask them to register how many of the eligible patients did not participate.

### Psychometric properties

In this result section, we will first discuss the acceptance rates of the questionnaire items and their reliabilities. Then the dimensional structures are discussed, followed by the convergent validation using the CPS_post_ item.

#### Acceptance and internal consistency

Completion rates of the patient version of the scale (SDM-Q-9) exceeded 95% for all items. Item difficulties were substantially above the midpoint of the 0–5 scale ranging from 3.5 to 4.3. Reliability analyses of the scale showed a high Cronbach’s α of 0.88. Corrected item-total correlations were substantial, i.e., above 0.40 (ranging from 0.43–0.75) except for Item 1 (0.38). Inter-item correlations ranged from 0.16 to 0.78, with a mean of 0.44. The lowest correlation of 0.16 was between Item 5 and Item 7.

Similar results were found for the physician version of the scale (SDM-Q-Doc). Completion rates for the physicians exceeded 98% for all items. Item difficulties were above the midpoint of the 0–5 scale ranging from 3.3 to 4.5. Reliability analyses also showed a high Cronbach’s α of 0.87. Corrected item-total correlations were substantial, i.e. above 0.40 (ranging from 0.43–0.79) except for Item 9 (0.26). Inter-item correlations ranged from 0.08 to 0.70 with a mean of 0.41. The lowest correlation of 0.08 was between Item 3 and Item 9 (Table 3).

**Table 3:** Item Characteristics of the SDM-Q-9 and SDM-Q-Doc.

	Acceptance (completion rates in %)		Discrimination (corrected item-total correlations)		Difficulty (mean range 0–5)		Cronbach’s alpha if item deleted	
Item	SDM-Q-9	SDM-Q-Doc	SDM-Q-9	SDM-Q-Doc	SDM-Q-9	SDM-Q-Doc	SDM-Q-9	SDM-Q-Doc
**1**	98.4	99.1	0.38	0.43	3.7	3.9	0.88	0.87
**2**	96.7	99.5	0.58	0.63	3.5	3.25	0.87	0.86
**3**	96.2	98.6	0.66	0.69	3.5	3.6	0.86	0.85
**4**	96.7	99.1	0.70	0.73	3.7	3.6	0.85	0.85
**5**	95.6	100.0	0.63	0.47	4.2	4.1	0.86	0.87
**6**	95.6	99.5	0.73	0.73	3.6	3.6	0.85	0.85
**7**	96.7	99.5	0.71	0.79	3.5	3.4	0.85	0.84
**8**	95.6	98.6	0.75	0.70	3.7	3.6	0.85	0.85
**9**	97.3	99.1	0.43	0.27	4.3	4.5	0.88	0.88
**Scale**							0.88	0.87

#### Convergent validity

Since the Intra-class coefficient for the SDM-Q-9 was only 0.06, we did not use a hierarchical analysis.

**Fig 1:**
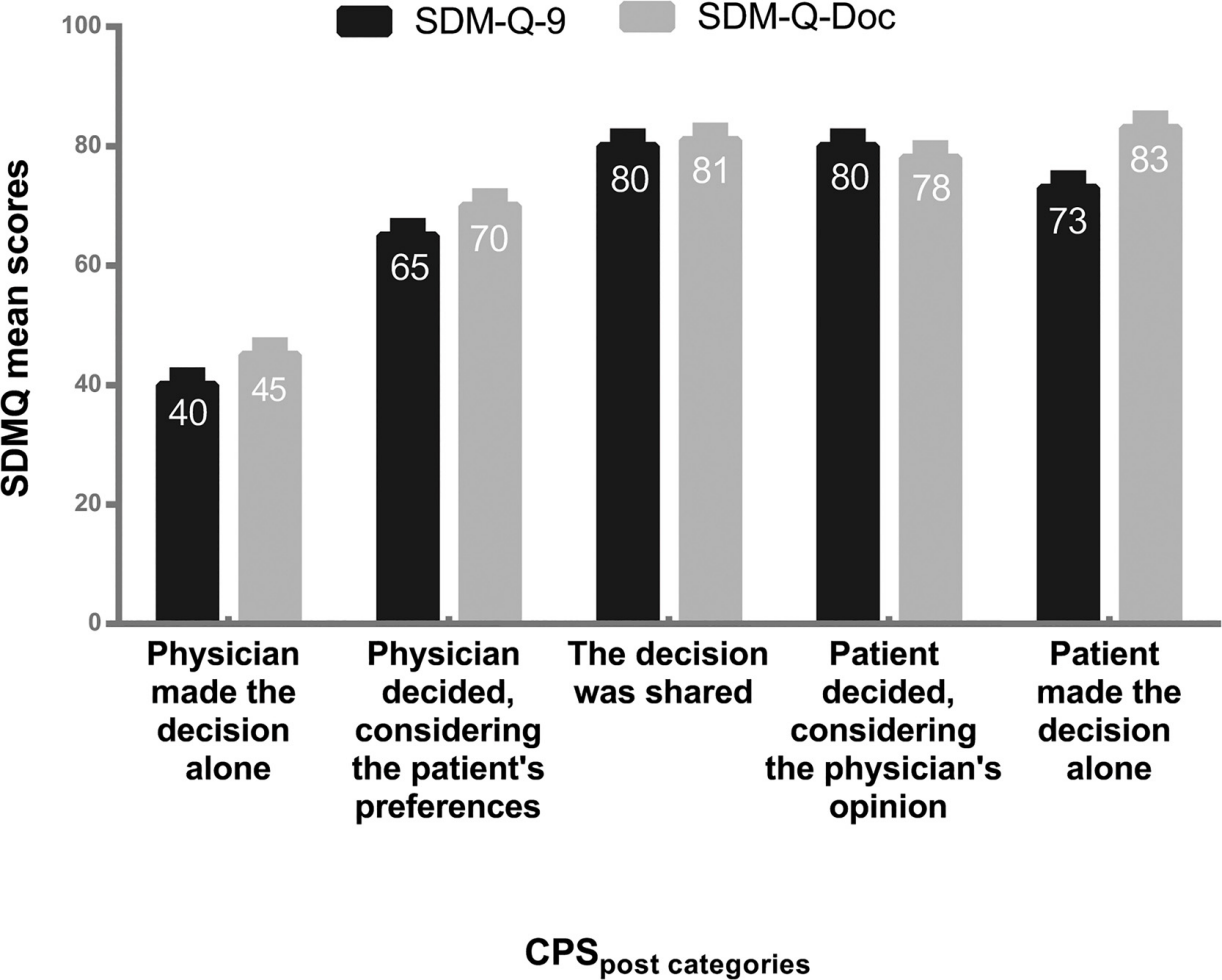
Mean SDM-Q-9 and SDM-Q-Doc scores by category of the CPS_post._

The SDM-Q-9 and SDM-Q-Doc total scores were significantly correlated with the CPS_post_ (*r* = 0.29 (SDM-Q-9) and *r* = 0.48 (SDM-Q-Doc), both *p*< 0.001). Fig 1 shows the mean SDM-Q-9 and SDM-Q-Doc scores per CPS_post_ categories for the two samples (Fig 1).

Notwithstanding the non-normality, the bootstrap analysis of both questionnaires showed that the violations of the normality assumption were of little influence on the detailed results. All confidence intervals and all tests came to essentially the same results, be it slightly more variable for the patient than for the physician questionnaire. All overall tests indicated that there were significant differences in total scores of both the SDM-Q-9 and the SDM-Q-Doc between the CPS_post_ categories (Table 4). Overlapping intervals indicate non-significant differences between the means of the CPS_post_ categories. Table 5 shows the total SDM-Q-9/SDM-Q-Doc scale scores as well as the confidence intervals for the five categories of the CPS_post_ item (Table 5). As can directly be seen from the confidence intervals, all multiple comparison tests (both equal and unequal variances) indicated homogeneous subsets for the SDM-Q-9: (1,2,5) and (2,3,4,5) and for the SDM-Q-Doc (1), (2,4) and (3,4,5).

**Table 4:** Significance tests for the SDM-Q-9 and the SDM-Q-Doc using CPS_post_ categories.

	SDM-Q-9			SDM-Q-Doc		
Test	*df*	Value	*p*	*df*	Value	*p*
**ANOVA F**	(4,123)	8.8	<0.001	(4,155)	25.0	<0.001
**Welch test**	(4,38)	8.4	<0.001	(2,101)	20.9	<0.001
**Brown-Forsyth**	(4,60)	7.6	<0.001	(2,146)	27.2	<0.001
**Kruskal-Wallis**	4	22.1	<0.001	4	50.7	<0.001
**Median test**	4	11.9	0.018	4	30.6	<0.001
**Jonckheere-Terpstra test**	-	3.3	0.001	-	6.2	<0.001

**Table 5:** Means and confidence intervals for assessing the convergent validity of the SDM-Q-9 and SDM-Q-Doc using the CPS_post_.

	SDM-Q-9					
CPS_post_	N	Mean	*S*_*e*_	Bootstrap *S*_*e*_	Confidence Interval	Bootstrap Confidence Interval
**Physician decided**	11	39.4	7.2	7.1	23.3–55.5	26.1–55.2
**Physician decided, considering patient’s preferences**	25	64.9	5.4	5.5	53.7–76.0	54.0–75.5
**Shared decision**	55	81.1	2.6	2.6	75.9–86.3	75.8–85.8
**Patient decided, considering physician’s opinion**	20	80.1	3.3	3.3	73.2–87.1	73.7–86.4
**Patient decided**	17	72.5	8.0	7.7	55.5–89.5	56.9–87.2
**Total**	128	73.1	2.3	2.3	68.6–77.6	68.5–77.6
	**SDM-Q-Doc**					
**Physician decided**	19	44.7	3.9	4.0	36.4–52.9	36.9–52.4
**Physician decided, considering patient’s preferences**	44	69.0	2.5	2.6	64.0–74.1	63.7–73.7
**Shared decision**	55	80.6	1.7	1.7	77.1–84.1	77.2–84.0
**Patient decided, considering physician’s opinion**	24	77.5	3.0	3.1	71.2–83.8	71.5–83.3
**Patient decided**	18	82.6	2.4	2.4	77.4–87.7	77.8–87.5
**Total**	160	73.0	1.5	1.5	70.0–75.8	69.8–75.7

Thus, as expected, category 1 (“Physician decided”) had a significantly lower mean SDM-Q-9 score than categories 3 and 4 in the patient sample. The mean of category 2 (“Physician decided considering the patient’s preferences”) and category 5 (“Patient decided”) were not significantly different from any of the other categories.

**Fig 2:**
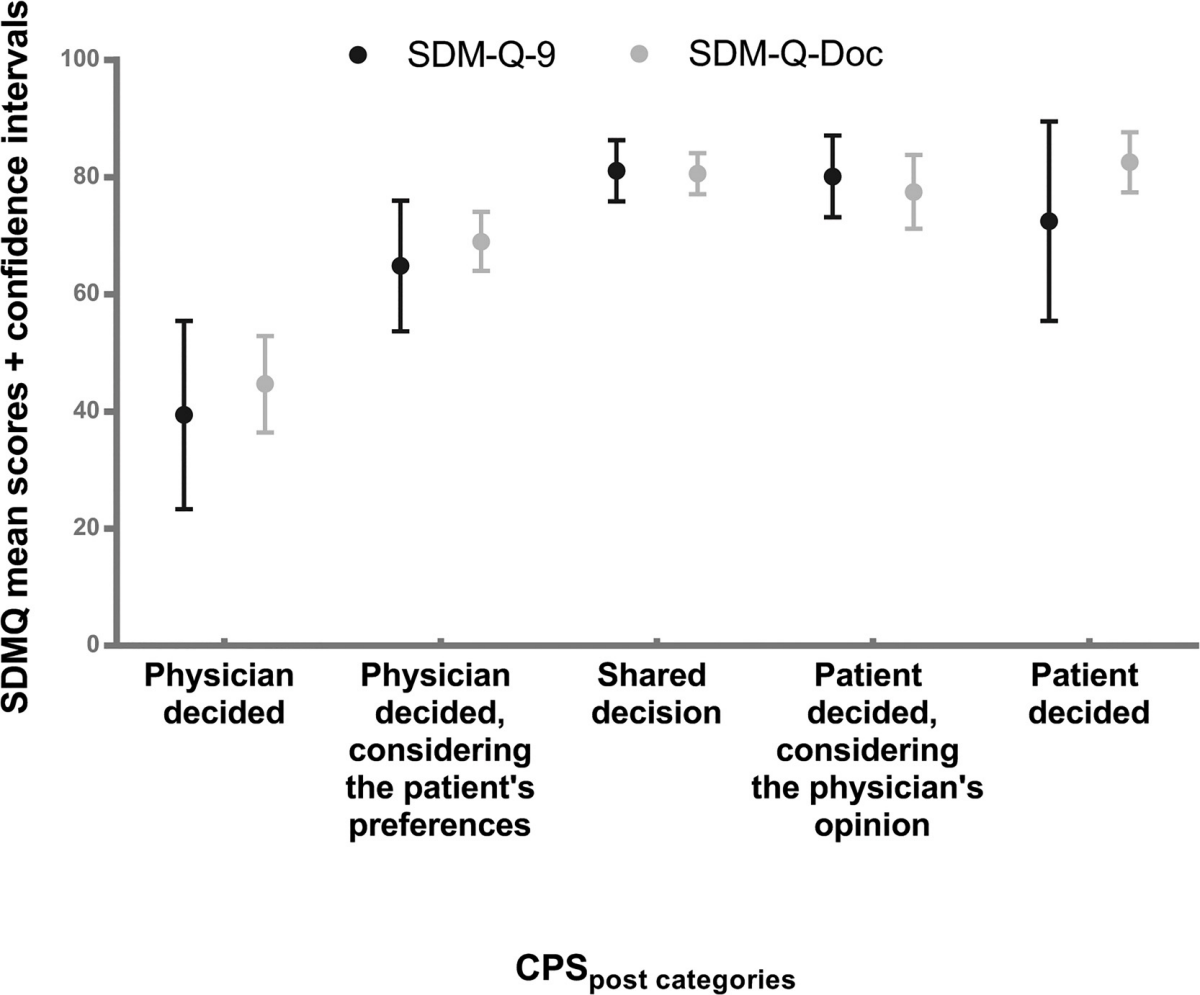
Mean scores and confidence intervals of the SDM-Q-9 and SDM-Q-Doc by category of the Control Preferences Scale.

For the SDM-Q-Doc the mean of category 1 (“Physician decided”) was significantly lower than the means of all other categories. The mean of category 2 (“Physician decided considering the patient’s preferences”) was significantly lower from that of category 3 (“Shared Decision”) and 5 (“Patient decided”). Categories 3, 4 and 5 were not significantly different from each other (Fig 2).

#### Factor structure

To assess whether a single component would account for sufficient variance to confirm the original single scale based on the nine items, we carried out a principal component analysis (PCA). The Kaiser-Meyer-Olkin measures verified the sampling adequacy for the analyses: KMO was > 0.85 for both samples, and almost all KMO values for individual items were above 0.8 in both samples, except for Item 1 in the patient sample (0.67), which was still above the acceptable limit of 0.5. This means that the patterns of correlations are relatively compact and factor analyses should yield distinct and reliable factors [21]. Bartlett’s test of sphericity was significant for both scales, indicating sufficient correlations between the items for a factor analysis to be appropriate (χ^2^ (36) = 724, *p* < 0.001 (SDM-Q-9) and χ^2^ (36) = 795, *p* < 0. 001 (SDM-Q-Doc)). In both samples two components had eigenvalues over Kaiser’s criterion of 1.0, with component 1 explaining 51.4% of the variance for the SDM-Q-9 and the second component explaining 13.5%. For the SDM-Q-Doc the first component explained 50.1% of the variance, the second 12.4%. Inspection of the scree plots suggested a two-component solution in both samples. After oblimin rotation, items 3 through 9 of the SDM-Q-9 loaded reasonably to highly on the first component (range 0.45–0.94), items 1 and 2 had loadings < 0.4 on the first component (-0.14 and 0.22) and loaded highly on the second component (0.94 and 0.72). For the SDM-Q-Doc all items except three had high loadings on the first component. Item 9 had the lowest loading (-0.15), followed by Items 1 (0.14) and 5 (0.19). These items loaded reasonably high (range, 0.63–0.81) on the second component (Table 6). These results point to a possible second component for both forms of the questionnaire.

**Table 6:** Results of the Principal Factor Analysis (PCA) for the SDM-Q-9 and SDM-Q-Doc.

	SDM-Q-9 (patients)			SDM-Q-Doc (physicians)		
Item	Component 1	Component 2	h^2^	Component 1	Component 2	h^2^
**1**	-0.14	**0.94**	0.80	0.14	**0.65**	0.52
**2**	0.22	**0.72**	0.70	**0.73**		0.54
**3**	**0.45**	**0.47**	0.60	**0.86**		0.68
**4**	**0.81**		0.67	**0.75**	0.14	0.67
**5**	**0.67**	0.12	0.53	0.19	**0.63**	0.53
**6**	**0.85**		0.72	**0.89**	-0.10	0.74
**7**	**0.94**	-0.16	0.78	**0.85**		0.76
**8**	**0.90**		0.77	**0.73**	0.13	0.62
**9**	**0.46**	0.13	0.28	-0.15	**0.81**	0.58

Since the German SDM-Q-9 showed a one-dimensional structure based on a PCA and this was confirmed in the Spanish version of the patients’ scale and the German SDM-Q-Doc scale as well, we also conducted a confirmatory factor analysis (CFA) on our patient and physician data.

Based on the results of the other studies we tested: 1) the one-factor model (Model 1); 2) a one-factor model excluding Item 1(Model 2), because this model obtained the best fit in the Spanish SDM-Q-9 and Item 1 had low factor loadings and low corrected item-total correlations in both our samples; 3) a one-factor model excluding Item 9 (Model 3), because of the low corrected item-total correlations of Item 9 in both our samples, especially in the physician sample; and 4) a one-factor model excluding both Items 1 and 9 (Model 4).

Because our data followed a non-normal distribution, we used maximum likelihood (LM) as well as robust statistics in our analysis; the latter are resistant to errors in the results produced by deviations from assumptions (e.g., of normality) [22, 23].

Results showed that two cases in the physician sample accounted for too much of the kurtosis, so these were excluded from the analysis [22].

For the patients, Model 1 did not meet any of the cut-off criteria of the fit indices, and Model 2 yielded acceptable fit indices, meeting the cut-off criteria for two of them (RMSEA and SRMR) and improving the others (χ^2^ and CFI) (Table 7). The best indices of fit were obtained with Model 4. For the SDM-Q-Doc Model 1 only met the cut-off criterion of the SRMR. All other models had an acceptable fit, meeting the cut-off criteria of the CFI and the SRMR. However the best solution was obtained with Model 4 (Table 7).

**Table 7:** Results of the Confirmatory Factor Analysis (CFA) for the SDM-Q-9 and SDM-Q-Doc: Maximum Likelihood (ML) and Robust statistics.

	df	χ^2^		CFI		RMSEA		SRMR
		ML	Robust	ML	Robust	ML	Robust	ML
**SDM-Q-9**								
**Model 1**	27	137.4***	76.5***	0.84	0.88	0.16 (0.13–0.19)	0.11 (0.08–0.14)	0.09
**Model 2**	20	77.8***	41.9 *	0.91	0.94	0.14 (0.10–0.17)	**0.08** (0.05–0.12)	**0.06**
**Model 3**	20	106.8***	55.5***	0.88	0.91	0.17 (0.13–0.20)	0.11 (0.07–0.14)	0.09
**Model 4**	14	56.2***	27.4 *	0.93	**0.96**	0.14 (0.10–0.18)	**0.08** (0.03–0.12)	**0.06**
**SDM-Q-Doc**								
**Model 1**	27	74.8***	56.9***	0.94	0.93	0.09 (0.07–0.12)	0.08 (0.05–0.10)	**0.06**
**Model 2**	20	49.7***	35.2 *	**0.96**	**0.96**	0.09 (0.06–0.12)	**0.06** (0.03–0.09)	**0.05**
**Model 3**	20	57.9***	40.7 **	**0.95**	**0.95**	0.10 (0.07–0.13)	0.07 (0.04–0.10)	**0.05**
**Model 4**	14	33.9 **	**21.3**	**0.97**	**0.98**	0.08 (0.05–0.12)	**0.05** (0.00–0.09)	**0.04**

Recommended values: CFI (comparative fit index) >0.96, RMSEA (root mean square error of approximation) ≤ 0.06 and SRMR (root mean square residual) ≤0.08. Values meeting cut off criteria are in bold.

* *p*-value < .05

** *p*-value < .01

*** *p*- value < .001.

## Discussion and Conclusion

### Discussion

This study translated Shared Decision Making Questionnaire for patients (SDM-Q-9) and physicians (SDM-Q-Doc) into Dutch and describes the psychometric evaluation of both scales. Good acceptance, internal consistency, and acceptable-to-good convergent validity were demonstrated.

The Dutch SDM-Q-9 and SDM-Q-Doc have similar psychometric properties as the original instruments and the Spanish SDM-Q-9 and they showed somewhat higher acceptance than the original German version (> 80%- 88% (SDM-Q-9) and > 93% (SDM-Q-Doc)).

Based on the literature [17] a Cronbach’s α coefficient higher than 0.7 is desirable, which was the case for both versions. Corrected item-total correlations were lower than the original German patient version (corrected-item-total scores >.7), but in accordance with the Spanish version of the SDM-Q-9. The Spanish study also found a low corrected item-total score for Item 1 (“My physician / I made clear to me/my patient that a decision needs to be made”), meaning that this item correlates poorly with the other items. In our study, the item-total correlation of Item 9 (“My physician and I reached an agreement on how to proceed”) of the patient version, was also relatively low compared to those of the other items, but this was not seen in the German and Spanish versions of the SDM-Q-9.

Corrected-item-total scores of the Dutch SDM-Q-Doc are similar to those of the original German version, with lowest scores on Items 1 and 9. The discrimination of 0.27 of Item 9 of the Dutch scale was much lower than that of the German version (0.44).

Besides the four limited investigations regarding the validity of the SDM-Q-9 and SDM-Q-Doc, mentioned in the Introduction, there has been little further study of the validity of the scales. Convergent validity of the SDM-Q-9 was tested by Scholl and colleagues (2012) [12] by comparing it to an observer rating scale measuring the extent to which physicians involve patients in decision making, the OPTION (observing patient involvement) scale, but no substantial correlation was found and convergent validity of the SDM-Q-9 could not be established. In a study on the interrelatedness of SDM measures, Kasper et al. [13] could not find any significant correlation between the same OPTION scale and the SDM-Q-9, but did find a moderate correlation between the SDM-Q-9 and their CPS_post_. Perception of a more autonomous role of the patient (CPS_post_) was associated with more involvement as reported on the SDM-Q [12]. In line with Kasper et al.’s findings, we also found a significant albeit low correlation of the SDM-Q-9 (*r* = 0.29) and a significant moderate correlation of the SDM-Q-Doc (*r* = 0.48) with the CPS_post_. The shared category of the CPS_post_ is in the middle, therefore an association of the SDM-Q-9 /SDM-Q-Doc with the CPS_post_ would not be linear, according to our hypothesis, which makes the interpretation of a Spearman’s correlation coefficient difficult. Still, in case of non-linearity (SDM-Q-9), a significant Spearman’s correlation coefficient tells us that an association exists. Furthermore we did not expect very strong associations, since the scales do not exactly measure the same construct: the SDM-Q-9/SDM-Q-Doc measures the process of SDM and the CPS_post_ item only assesses the final decision. However our results do support the hypothesis that the scales are related, especially when we look at the categorical analyses. Patients and physicians both had the lowest scores on the SDM-Q when they felt the physician had decided. In addition, patients also had the highest SDM-Q scores when they perceived their consultations as more shared as based on the CPS_pos_. For physicians SDM-Q-scores were highest when the patient decided alone or together with the physician, likely reflecting that an active role of the patient fits a shared process more than a consumerist or informed process, because in general participation of patients is low. The differences between the other categories on the CPS (2,4,5) are less clear and seem to carry different meanings for patients and physicians. More research on this topic might provide insight into what SDM actually means to different stakeholders.

Results of our PCA and CFA were similar to the findings of De Las Cuevas et al. [11]. In our study, the PCA yielded a two-component solution for the SDM-Q-9, with factor loadings above 0.6 on the second component for Items 1 and 2. In addition, for the SDM-Q-Doc Items 1, 5 and 9 showed low loadings on the first component and loaded above 0.6 on the second component. The PCA on the Spanish version of the SDM-Q-9 also showed a two-component solution, with factor loadings above 0.5 on the second component for the same items, Items 1 and 2. Furthermore a CFA showed that the best solution was obtained for a one-factor model without Item 1 [11]. For the SDM-Q-Doc results of our CFA were comparable to the German version. For the original German SDM-Q-Doc, a CFA showed factor loadings of Items 1 and 9 below 0.4, but the items were retained based on their coherence with theory of the SDM construct [9].

Although the psychometric results of our study on the Dutch SDM-Q-9 and SDM-Q-Doc are largely concordant with the results of the original German scales, and the scales thus are suitable for use in a primary and specialised hospital care samples, there are some differences. Incongruity between the SDM-Q-9 questionnaires might be explained by factors like age and gender, since our sample consisted of relatively older patients (mean age of 61 years old) and a slightly smaller percentage of women (60%) compared to the samples in the Spanish study (mean age 45 years and 70% of women). We also had younger physicians (34 years of age) compared to the German physician sample (50 years of age) [6,8,9]. Differences might also be caused by the fact that we chose for less strong statements in some items, following the English version, or by cross-cultural differences between Germany and the Netherlands regarding the physician-patient relationship. However, in case of translation problems, the difficulties with certain items should have been the same in both Dutch versions (SDM-Q-9 and the SDM-Q-Doc), but this was not the case.

The main strength of this study is the fact that we examined both the patient (SDM-Q-9) and the physician versions (SDM-Q-Doc) of the 9-item Shared Decision Making Questionnaire in the same diverse clinical settings. However, since this was not the case for the original German- and the Spanish validation studies, comparison of the Dutch versions with (and between) the other versions is difficult. The fact that our sample consisted of physician and patient groups in primary and specialised hospital (secondary) care, could have played an important role in the differences we found.

These differences may also reflect the discussion in the literature on the conceptualization of SDM. There is no general consensus yet between different parties on what constitutes the SDM-process [24, 25]. The SDM-Q-9 was based on theoretically defined steps of physician behaviours in a shared decision making process [6]. Possibly, Item 1 does not necessarily relate to a role of the patient in making the decision. That is, the physician could make explicit that a decision needs to be made but implicitly assume that it is the role of the physician to make that decision, which might especially be the case in specialised care. Furthermore Items 1 and 9 may be seen not as part of the shared decision making process itself, but as facilitators to engage patients in (Item 1) or to conclude the process (Item 9).

The psychometric results of the original SDM-Q-Doc were similar to the results for both our SDM-Q-9 and SDM-Q-Doc. All of these scales were tested in both a primary and a secondary care sample (both including Type 2 diabetic and psychiatric (depression) health problems) and found that Item 9 (“My patient and I reached an agreement on how to proceed”) had low corrected item-total correlations [8] with the scale scores. This could mean that this aspect of SDM is less relevant or more complicated in specialised care or even different for specific specialties, which was also found in another study of De Las Cuevas and colleagues [26]. Their findings suggest that the process to come to a shared decision may have a distinctive profile, depending on the type of diagnosis/health problem [26]. More research on the German, Spanish and Dutch scales together and comparison of the different study populations would may shed light on this. Such research can be useful to improve the instruments and will tell us more on the shared decision making concept, between patients and physicians, in different cultures and different specialism. In addition, mixed methods research can provide insight in how patients, physicians and observers view SDM and will help further define what SDM is at its core.

The main limitation of this study is the fact that there are no comparable instruments, which are necessary for the proper assessment of the convergent validity.

Even though the total sample sizes were sufficient for our analyses, we did not recruit enough participants of every specialty to compare mean scores or test for differences in internal consistency between primary and specialized care or the different medical specialities.

Another limitation is the fact that we included a convenience sample of patients with different backgrounds. We have no information on non-participants and cannot evaluate if and if so, to what extent, participant selection may have biased our results.

Finally there were potential differences in procedures between centres. All participating departments received the same instructions, information, and questionnaires, but we cannot verify if participating physicians complied with the instruction to complete the questionnaire immediately after consultation. Delayed responses could have led to recall errors and imprecise ratings [8], but this was also true for the procedures in the other countries.

### Conclusion

Taken together, our findings suggest that the Dutch versions of the SDM-Q-9 and SDM-Q-Doc questionnaires are suitable for use in Dutch primary and specialised care. The scales are acceptable to participants, demonstrate good reliability and are related as expected with the CPS_post_.

The questionnaires show an acceptable (patients) to good (physicians) model fit with a one-dimensional structure. Results of this validation study call into question Item 1, and Item 9, and thus the concept used. However we believe these items address important aspects of SDM. Based on the coherence with theory and comparability with other studies, we therefore suggest keeping all nine items of the scale, until more light is shed on this issue. Still further testing of the validity of the questionnaires in different clinical settings and more research on the concept of SDM is necessary.

## Supporting Information

S1 Appendix Item content of the Shared Decision Questionnaire for Patients (SDM-Q-9) and Physicians (SDM-Q-Doc) in English. For Dutch version, please email a.m.stiggelbout@lumc.nl.
(DOCX)
