# Supplementary material for: Dutch Translation and Psychometric Testing of the 9-Item Shared Decision Making Questionnaire (SDM-Q-9) and Shared Decision Making Questionnaire-Physician Version (SDM-Q-Doc) in Primary and Secondary Care
Source: PLoS One. 2015 Jul 7;10(7):e0132158. doi: 10.1371/journal.pone.0132158 (PMC4494856; doi:10.1371/journal.pone.0132158)
Supplement: S1 Appendix — For Dutch version, please email a.m.stiggelbout@lumc.nl. (DOCX) [file pone.0132158.s001.docx]

| SDM-Q-9 (a) / SDM-Q-Doc (b) |
| --- |
| 1a. My physician made clear that a decision needs to be made.  1b. I made clear to my patient that a decision needs to be made. |
| 2a. My physician wanted to know exactly how I want to be involved in making the decision.  2b. I wanted to know exactly from my patient how he/she wants to be involved in making the decision. |
| 3a. My physician told me that there are different options for treating my medical condition.  3b. I told my patient that there are different options for treating his/her medical condition. |
| 4a. My physician precisely explained the advantages and disadvantages of the treatment options.  4b. I precisely explained the advantages and disadvantages of the treatment options to my patient. |
| 5a. My physician helped me understand all the information.  5b. I helped my patient understand all the information. |
| 6a. My physician asked me which treatment I prefer.  6b. I asked my patient which treatment option he/she prefers. |
| 7a. My physician and I thoroughly weighed the different treatment options.  7b. My patient and I thoroughly weighed the different treatment options. |
| 8a. My physician and I selected a treatment option together.  8b. My patient and I selected a treatment option together. |
| 9a. My physician and I reached an agreement on how to proceed.  9b. My patient and I reached an agreement on how to proceed. |
| After the translation and validation of the SDM-Q-9 and SDM-Q-Doc we received some comments on the answering categories from researchers who were interested in using the scales. The response choices in the Dutch version were comparable to those in the original German version: we used the original answering categories ”does not completely apply” to “does apply completely” (in German ”Überhaupt nicht zutreffend ” to “Ganz zutreffend”). However, in Dutch, “does not apply” (translated ”niet van toepassing”) is a category that is often used in questionnaires for items one can skip, because they do not apply to that person or situation. Since this could create difficulties for some patients, we suggest the use of the answering categories comparable to the official English version, ”completely agree” to ”completely disagree”. |
